# The burden experienced by family caregivers of patients with epilepsy attending the government psychiatric hospital, Kaduna, Nigeria

**DOI:** 10.4314/pamj.v5i1.56176

**Published:** 2010-06-01

**Authors:** Folorunsho Tajudeen Nuhu, Abdulkareem Jika Yusuf, Akinsola Akinbiyi, Joseph Oluyinka Fawole, Obafemi Joseph Babalola, Zainab Titilope Sulaiman, Olaniyi Oyeniran Ayilara

**Affiliations:** 1 Federal Neuropsychiatric Hospital, Barnawa, Kaduna. Nigeria; 2 Department of Psychiatry, Ahmadu Bello University Teaching Hospital, Zaria, Nigeria; 3 Mercy Mental Health 117-129 Warring Crescent, Horpers crossing 3029 Australia; 4 Department of Psychiatry, National Hospital, Abuja. Nigeria

**Keywords:** Disease burden, Caregiver, Epilepsy, Patients, Kaduna, Nigeria

## Abstract

**Background:**

Caring for patients with chronic medical and psychiatric disorders is associated with significant burden. However little is known about the burden experience by caregivers of patients with epilepsy in Nigeria. The objective of this study, therefore, was to assess the level and correlates of burden among caregivers of patients with epilepsy.

**Methods:**

It was a cross-sectional study carried out among 231 eligible caregivers of patients with epilepsy attending the psychiatric clinic of government psychiatric hospital in Kaduna, Northern Nigeria. Sociodemographic/clinical characteristics of patients and socio-demographic characteristics of caregivers were recorded, and the Zarit Burden Interview administered to caregivers to assess their experience of burden.

**Results:**

The mean age of the caregivers was 43.6 ±9.5 years, 52.4% lived outside Kaduna and the mean seizure-free period for the patients was 26.4 ±36.5 weeks. One hundred and twenty (51.9%) caregivers had high burden. High burden was significantly associated with patients aged less than20 years, patient’s unemployment, long duration of epilepsy, short seizure-free period, family history of epilepsy and living outside Kaduna (p value<0.05).

**Conclusion:**

Caregivers of patients with epilepsy experience significant burden while caring for their relatives and this is mainly associated with patient’s factors and location of residence. Therefore efforts should be made control seizure and make health care available and affordable to all citizens irrespective of where they live.

## Background

Burden of care is a multi-factorial construct which includes emotional, psychological, physical and economic impact as well as related distressing feelings such as shame, embarrassment, anger, feeling of guilt and self-blame [1]. It is customary to describe burden as objective or subjective. Objective burden refers to changes in household routine, family or social relations, work, leisure and physical health; while subjective burden consists of subjective distress among relatives, including impact on mental health [2].

Family caregivers have been described as forgotten patients and it was suggested that caregiver’s symptoms such as mood swing, fatigue, headaches, joint and muscle pains, marital and family conflicts, and financial problems may be a reflection of caregiver stress in looking after a sick relative [3].

Studies have shown that caregivers of patients with epilepsy have high levels of strains, fears that the illness may cause injury or death as well as concern about what will happen to patients in future when the caregiver will not be available to cater for patients [4, 5]. In addition, it has been shown that relatives who care for patients with epilepsy have higher burden of care than control groups [6-8] and that depression and patient’s functioning separate from seizure control [6] and low income [7] are predictors of burden in caregivers. In Nigeria, it has been reported that caregivers of patients with schizophrenia [9-11] and dementia [12,13] are strained while caring for their relatives and that high burden was associated with living in rural areas [9-11], large family size [11], severity of patient’s illness and caregiver’s low level of education [13]. However there is paucity of literature on the burden experienced by caregivers looking after patients with epilepsy especially in the northern part of Nigeria and it is against this background that we studied caregivers of epileptic patients in our centre to assess their level of burden in caring for their patients.

## Methods

This cross-sectional study was carried out among primary caregivers of patients with epilepsy attending the outpatient clinic of the Federal Neuropsychiatric Hospital, Kaduna in the North-western part of Nigeria. Kaduna, the capital of Kaduna State is about 200km from the Federal Capital Territory, Abuja. The hospital receives referral from all states in the Northern Nigeria. Some patients and relatives travel for about 300-350 km to access our health care facility.

All consecutive primary caregivers who have been caring for the patients with epilepsy, for at least one year, seen in the outpatient clinic between March, 2008 and September, 2008 were recruited for the study excluding relatives whose patients had any of the following; comorbid chronic psychotic or affective disorders, other chronic physical disorders apart from epilepsy, cognitive disorder such as mental retardation or dementia.

The socio-demographic/clinical characteristics of the patients and socio-demographic variables of the caregivers were recorded. The clinical information such as duration of illness, seizure-free period (calculated from the day of last seizure to the time of interview), past medical and psychiatric history as well as family history of epilepsy were obtained from the case files. In addition we conducted mental state assessment and physical examination on the patients. We administered the Zarit Burden Interview (ZBI) instrument [14] to all the eligible caregivers. The ZBI is a 22-item questionnaire with each item rated from 0-4 (higher scores denoting higher burden for a particular item).

The test-retest reliability and face validity of ZBI has been established in Nigeria [13] and the instrument has previously been used in some studies conducted in the country [11,13]. Few caregivers who did not understand English language were interviewed by one of the authors (ZTS) using the Hausa version obtained through the method of back-translation [15]. The total burden for a subject is the sum of the scores in all the items. We calculated the median score for all the subjects which is the midpoint between the lowest total score and the highest total score of the burden interview. The median is a measure of central tendency not sensitive to outlying values. Subjects whose scores were below the median score were rated ‘low burden’ while those scoring equal and above the median score were assessed to be ‘high burden’ [11,16].

The Research and Ethics committee of the Federal Neuropsychiatric Hospital, Kaduna gave approval for the study and all the participants gave informed consent.

## Statistical analysis

Using the 15^th^ edition of the Statistical Package for Social Sciences we calculated the frequencies and percentages for the variables. Chi-square(X^2^) test was used to determine the association between burden and categorical variables while logistic regression analysis was performed to determine the predictors of high burden. The level of significance was set at P value less than 0.05.

## Results

Table 1 and Table 2 shows the socio-demographic/clinical characteristics of caregivers and patients. For the caregivers the mean age was 43.6 ±9.5 years, mean year of education was 9.7 ±6.0 and mean income was US$116 ±827. For the patients; the mean age was 28±13.2, mean duration of illness was 9.5 ±8.2 years and mean seizure-free period was 26.4 ±36.5 weeks.

The median ZBI score was 25. One hundred and twenty (51.9%) caregivers scored 25 and above and were rated as having “high burden”.

The mean and standard deviation of each item of the ZBI score for all the caregivers are shown in Table 3. The highest mean burden score was found on item 7 (caregiver’s fear of what the future holds for the patients (2.56±1.12)). This was followed by item 13 (feeling uncomfortable about having friends or visitors around because of relative (1.82±1.76)).

Caring for patients below the age of 20 years (p value, 0.036), patient’s unemployment (p value, 0.024), long duration of illness (p value, 0.009), short seizure-free period (p value<0.001), family history of epilepsy (p value, 0.024) and living outside Kaduna (p value <0.001) were all significantly associated with caregiver high burden (Table 4). The other socio-demographic characteristics of caregivers were not significantly associated with high burden (p value>0.05).

After a logistic regression analysis the following factors were predictive of high burden in caregivers: seizure duration (p value=0.010, OR=2.634, 95%CI=1.264-5.487), seizure-free period (p value=0.002, OR=0.257, 95%CI=0.107-0.617) and location of residence (p value <0.001, OR=3.069, 95%CI=1.697-5.550)

**Table 1:** Socio-demographic/clinical characteristics of caregivers

**Variables**	**Frequency (N)**	**Percentage (%)**
**Age**		
Younger than 40 years	76	32.9
40 years and over	155	67.1
**Sex**		
Male	107	46.3
Female	124	53.7
**Years of education**		
Less than 6 years	53	22.9
6-12 years	92	39.8
Higher than 12 years	86	37.3
**Relationship to patients**		
Father	56	24.2
Mother	89	38.5
Spouse	41	17.7
Child	16	6.9
Others	29	12.7
**Employment status**		
Employed	192	83.1
Unemployed	39	16.9
**Financial support**		
Yes	112	48.5
No	119	51.5
**Income**		
Less than US$67 per month	44	19.0
US$67-134 per month	116	50.2
Greater than US$134per month	71	30.8
**Family size**		
5 or less	82	35.5
More than 5	149	64.5
**Location of residence**		
Within Kaduna	110	47.6
Outside Kaduna	121	52.4

**Table 2:** Socio-demographic/clinical characteristics of patients

Variables	Frequency (N)	Percentage (%)
**Age**		
Younger than 20 years	68	29.4
20-40 years	124	53.7
**40 years and over**		
Sex	39	16.9
Male	137	59.3
**Female**		
Duration of illness	94	40.7
1-5 years	91	39.4
6-10 years	69	29.9
**Longer than 10 years**		
Seizure-free period	71	30.7
Less than 6 months	161	69.7
6 months – 1 year	33	14.3
Longer than 1 year	37	16
Employment	105	45.5
**Employed**		
Unemployed	126	54.5

**Table 3:** Mean scores and standard deviation (SD) of items in the ZBI instrument

**Variables**	**Mean(SD)**
Feeling that relative asks for more help than he/she needs	1.33 (0.96)
Having no time for self because of time spent caring for relative	1.48 (1.00)
Feeling stressed between caring for relative and meeting other responsibilities	1.34 (0.87)
Feeling embarrassed over relative’s behaviour	1.29 (0.97)
Feeling angry when around relative	1.00 (0.87)
Feeling relative affect caregiver’s relationship with others negatively	0.90 (0.81)
Being afraid of what future holds for relative	2.56 (1.12)
Feeling that relative depends on the caregiver	1.32 (1.21)
Feeling strained when around relative	1.05 (0.89)
Caregiver feeling his/her health has suffered because of involvement with relative	0.75 (0.50)
Caregiver feeling he does not have enough privacy because of relative	1.40 (1.02)
Caregiver feeling his/her social life has suffered because of caring for relative	1.24 (0.85)
Feeling uncomfortable about having visitors because of relative	1.82 (1.76)
Feeling relative expect care as if caregiver is the only one to be depended on	1.04 (0.91)
Feeling no enough money to care for relative in addition to other expenses	1.10 (0.89)
Caregiver feeling he/she will be unable to care for relative much longer	0.96 (0.45)
Feeling loss of control of life since the onset of relative’s illness	0.87 (0.77)
Wishing to leave the care of relative to someone else	0.93 (0.91)
Feeling uncertain about what to do about relative	1.10 (0.85)
Caregiver feeling he/she should be doing more for relative	1.13 (0.93)
Caregiver feeling he/she could do a better job in caring for relative	1.07 (0.85)

**Table 4:** Burden and patients’ (and caregivers’) characteristics

	Low burden (N=111)	High burden (N=120)	X^2^	d/f	p value
**Age of patient**					
<20 years	26 (38.2)	42 (61.8)			
20-40 years	60 (48.4)	64 (51.6)	6.656	2	0.036
>40 years	25 (64.1)	14 (35.9)			
**Patient employment status**					
Employed	59 (56.2)	46 (43.8)	5.108	1	0.024
Unemployed	52 (41.3)	74 (58.7)			
**Duration of illness**					
1-5 years	55 (60.4)	36 (36.9)			
6-10 years	26 (37.7)	43 (62.3)	9.523	2	0.009
Longer than 10 years	30 (42.3)	41 (57.7)			
**Seizure-free period**					
Less than 6 months	62 (38.5)	99 (61.5)			
6 months- 1 year	22 (66.7)	11 (33.3)	19.660	2	<0.001
Longer than 1 year	27 (72.9)	10 (27.1)			
**Family history of epilepsy**					
Yes	28 (37.3)	47 (62.7)	5.111	1	0.024
No	83 (53.2)	73 (46.8)			
**Residential area**					
Within Kaduna	69 (62.7)	41 (37.3)	11.118	1	<0.001
Outside Kaduna	42 (34.7)	79 (65.3)			

## Discussion

The results of this study show that about 52% of our subjects experienced high level of burden while caring for their relatives and this was significantly associated with caring for younger patients (below 20 years of age) who were unemployed, had longer duration of illness, lived outside Kaduna, had family history of epilepsy and short seizure-free periods.

Majority of the caregivers are female and close to 40% are mothers. This is similar to a recent report among caregivers of patients with schizophrenia in Nigeria [11]. The cultural belief that men should work, and in most cases they are the bread-winners, may have shifted the responsibility of caring for the sick to the women-folk. One other possibility is the polygamous nature of most families in the study environment which makes it the duty of every mother to look after her own children.

More than half of the caregiver had high burden with their greatest concern being what will happen to the relatives in future when they (the caregivers) may not likely be available to look after them. However the low mean burden scores on items 16 and 18 (Table 3) suggest that caregivers will continue to care for their relatives despite their experience of burden. This is similar to the findings among caregivers of cancer patients in Southern Nigeria [17].

The association of caring for patients below the age of 20 and high burden was inconsistent with Martyns-Yellowe report [9] among caregivers of schizophrenic patients. He found that those who cared for patients in the age range “21-45” years were the most burdened. It is possible that caring for younger patients may be more stressful. This is the stage when children and adolescents are expected to be in school and especially if seizure is poorly controlled they may spend time out of school. In this instance caregivers may be forced to stay at home with patients. A study conducted in Nigeria has reported a significant impact of epilepsy on some parents caring for their children with the disease and a significant proportion of those parents had to stop working in order to have enough time to look after the children [18]. In addition children may not appreciate the effects of their illness on the whole family and they are more likely to be unemployed and have to depend on the caregivers for virtually all their needs.

Indeed, in this study unemployment was also found to be associated with high burden. In patients who have had the illness for a long time, the burden experienced by caregivers may be the cumulative effects of the disease over the years. A short seizure-free period may be an indication of severe illness. Repeated seizure attacks may challenge the coping ability of the caregiver. This may also involve frequent hospital visits, using high doses of anticonvulsant drugs (and hence increase cost), close monitoring of patients at home (with less time for other responsibilities), fear of receiving visitors because of stigma and fear of what future holds for the patient. Any of these (if present) may explain the burden experienced by the caregivers. Where there is family history of epilepsy, the burden experienced by caregivers may be the total burden of caring for two or more “sick relatives”, the burden associated with stigma, inability to meet financial requirement or a combination of all the above.

Caregivers living outside Kaduna had higher burden in this study. This is similar to previous reports among caregivers of patients with schizophrenia in Nigeria who lived in rural areas [9-11]. Indeed, rural dwellers are more likely to be poorer than their urban counterparts [9] and they have poor access to medical and mental health care. Poor access may result from poor road network, distance from centres where the health facilities are located or poverty [19]. In this study however, living outside Kaduna does not equate to living in a rural setting. The main factor is the distance patients and their caregivers have to cover before arriving at our hospital. Some of our subjects travel more than 300km with their patients to attend clinics in our centre, some travels a day before the clinic appointment and some have to sleep in Kaduna after the clinic to avoid night journey back home. On the whole caregiver may lose up to three days to ensure that their relatives keep a particular clinic appointment. This, no doubt, will worsen their financial constraints and increase the overall burden. Further analysis however showed that long duration of illness, short seizure-free period (poor seizure control) and living outside Kaduna are the main predictors of high burden in caregivers of patients with epilepsy. The effects of high burden can be enormous. In fact, it has been established that caregivers who experience high burden are at risk of developing emotional disorders such as depression [20,21].

## Limitations

It is a cross-sectional study carried out in only one centre in the country. We also excluded caregivers of patients with co-morbid medical or psychiatric disorders. However this was done to be certain that the burden experienced by caregivers actually resulted from caring for relatives with epilepsy and not other disorders. In addition, variables like duration of weekly or daily contact with the patients were not assessed.

## Conclusion

Caring for patients with epilepsy is really challenging and it is associated with enormous burden. Factors that predict high burden are long duration of epilepsy, poor seizure control and living far away from treatment centre. We therefore recommend that all efforts should be made to control seizure in epileptics and to make health care readily available to the entire populace at affordable price. Assistance could also come through the proper implementation of the National Health Insurance Scheme (NHIS).

## Competing interests

We declare that there is no competing interest.

## Authors’ contributions

FT Nuhu developed the idea and initiated the design of the study, took part in the data collection, data analysis and writing of the paper, and he read and approved the final copy.  AJ Yusuf took part in the design, data analysis and writing of the paper. He read and approved the final copy. Dr A Akinbiyi contributed to the design, data analysis and writing of the paper. He also read and approved the final copy. JO Fawole contributed to the design, data collection and writing. He read and approved the final copy. OJ Babalola, ZT Sulaiman and OO Ayilara contributed to the design, data collection, writing and approval of the final copy.
